# CSB ablation induced apoptosis is mediated by increased endoplasmic reticulum stress response

**DOI:** 10.1371/journal.pone.0172399

**Published:** 2017-03-02

**Authors:** Manuela Caputo, Alessio Balzerano, Ivan Arisi, Mara D’Onofrio, Rossella Brandi, Silvia Bongiorni, Stefano Brancorsini, Mattia Frontini, Luca Proietti-De-Santis

**Affiliations:** 1 Unit of Molecular Genetics of Aging—Department of Ecology and Biology—University of Tuscia, Viterbo, Italy; 2 Genomics Facility, European Brain Research Institute (EBRI) “Rita Levi-Montalcini”, Rome, Italy; 3 Department of Experimental Medicine—Section of Terni, University of Perugia, Terni, Italy; 4 Department of Haematology, University of Cambridge, Cambridge Biomedical Campus, Cambridge, United Kingdom; 5 National Health Service (NHS) Blood and Transplant, Cambridge Biomedical Campus, Cambridge, United Kingdom; 6 British Heart Foundation Centre of Excellence, University of Cambridge, Cambridge Biomedical Campus, Cambridge, United Kingdom; Duke University School of Medicine, UNITED STATES

## Abstract

The DNA repair protein Cockayne syndrome group B (CSB) has been recently identified as a promising anticancer target. Suppression, by antisense technology, of this protein causes devastating effects on tumor cells viability, through a massive induction of apoptosis, while being non-toxic to non-transformed cells. To gain insights into the mechanisms underlying the pro-apoptotic effects observed after CSB ablation, global gene expression patterns were determined, to identify genes that were significantly differentially regulated as a function of CSB expression. Our findings revealed that response to endoplasmic reticulum stress and response to unfolded proteins were ranked top amongst the cellular processes affected by CSB suppression. The major components of the endoplasmic reticulum stress-mediated apoptosis pathway, including pro-apoptotic factors downstream of the ATF3-CHOP cascade, were dramatically up-regulated. Altogether our findings add new pieces to the understanding of CSB mechanisms of action and to the molecular basis of CS syndrome.

## Introduction

Apoptosis evasion is a fundamental hallmark used by cancer cells to evolve resistance to cell death induced in response either to the intrinsic perturbation of metabolic circuitries or to anti-cancer drug therapies [1]. The ability to evade apoptosis is caused by a range of different alterations including the over-expression of anti-apoptotic factors, which bring back to sub-lethal levels the stress conditions associated to cancer cell metabolism [2]. Suppression of these anti-apoptotic factors might re-sensitize cancer cells to apoptosis and offer new therapeutical strategies in alternative or in association with conventional chemotherapy.

CSB is a member of the SWI/SNF ATP-dependent chromatin remodelling protein family that can wrap DNA and remodel chromatin [3–5]. Mutations in the *csb* gene lead to Cockayne syndrome (CS), a rare human autosomal recessive disorder characterized by a progressive degeneration of a wide range of tissues and organs and features of premature aging [6,7]. CSB participates in a number of different functions of a cell metabolism. It plays a role in transcription-coupled repair (TCR), an important sub-pathway of nucleotide excision repair (NER) that rapidly removes bulky DNA lesions located on the transcribed strand of active genes [8]. In addition, CSB plays a role during basal and activated transcription by stimulating all three classes of nuclear RNA polymerases [4,9,10]. Finally, CSB has been demonstrated to be a key regulator of p53, stimulating its ubiquitination and degradation, therefore re-equilibrating the physiological response toward cell proliferation and survival rather than cell cycle arrest and cell death upon stress [11–13]. Along these lines we speculated that CSB functions as an anti-apoptotic factor [14].

In a recent study, we demonstrated that a number of cancer cell lines from different tissues display dramatically increased expression of CSB protein and that its ablation induces massive cell death via apoptosis [15]. Interestingly, normal and cancer cell lines displaying normal expression of CSB remained unaffected when this protein was suppressed. The fact that CSB ablation specifically affects tumor cells, without harming non-transformed cells, suggests that the former are addicted to elevated levels of CSB. Here we investigated the effects on global gene expression in HeLa cells, caused by CSB silencing, in order to elucidate the molecular mechanism by which CSB depletion causes massive apoptosis in cancer cells.

## Results

### Microarray gene expression profile of Hela cells suppressed for CSB protein

**Fig 1:**
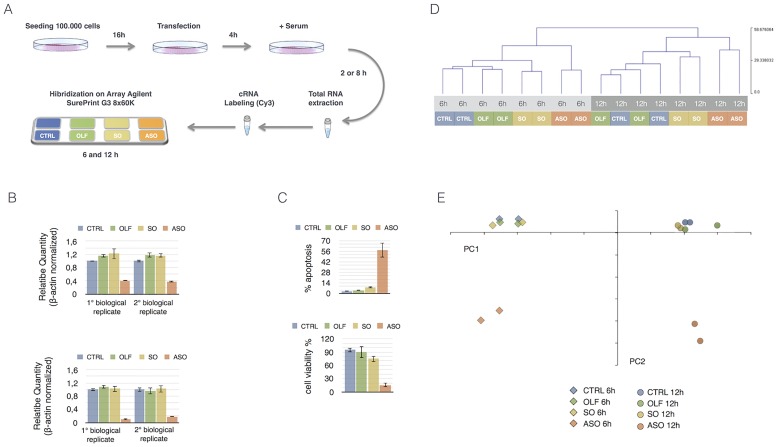
A) Experimental design: briefly cells were seeded at the confluence of 1X10^5^ cells and transiently transfected 16 h later with ASO or SO or simply exposed to the transfectant reagent (OLF). Cells were recovered 2h or 8h (6h and 12h after the start of the transfection) after the adding of the serum. Total RNA was used as starting material to generate labeled cRNA for successive array hybridization. Two biological samples for each experimental point were analyzed. B) Graphs showing qRT-PCR analysis of CSB mRNA expression, at 6h (upper panel) and 12h (lower panel) after the transfection for both the biological replicates. The results, normalized to β-actin, were averaged from values obtained by performing three technical replicates. The values are means ± SD. C) Graphs showing apoptosis and cell viability percentage 48h after CSB ablation. The results were averaged from values obtained by performing three technical replicates. The values are means ± SD. D) Hierarchical clustering of samples, obtained using a selection of all filtered: 17703 probes that have a |Dev.st/Mean = CV|<5% in Log2 expression values between biological replicates, across all samples. E) Principal Component Analysis of samples, using a selection of all filtered: 17703 probes that have a |Dev.st/Mean = CV|<5% in Log2 expression values between biological replicates, across all samples.

We have recently shown that CSB ablation induces a pro-apoptotic effect [15]. To gain insights into the mechanisms underlying this pro-apoptotic effect we performed genome wide gene expression analysis. For this purpose, cells were incubated with antisense oligonucleotides specifically inducing CSB mRNA degradation (ASO). Moreover, we used three different experimental controls: mock treated cells (CTRL), transfection reagents alone (OLF) and cells treated with sense oligonucleotides (SO). Two biological replicates were performed for each experimental point and two time points (6 and 12 h) were chosen in order to study the early perturbations in the transcriptome arising from the suppression of CSB (Fig 1A). Relative CSB expression was verified by qRT-PCR, for each experimental point, as shown in Fig 1B. A reduction of around 60% (std 0.02) and 80% (std 0.015) in CSB mRNA expression was detected at 6 and 12h, after transfection, respectively. As expected, suppression of CSB results in massive induction of apoptosis (57% of cells + 9.3 std) and a strong reduction of cell viability, at 48h post transfection (Fig 1C).

Global gene expression patterns were then determined using microarrays (Agilent SurePrint G3 Human GE 8x60K) representing the entire transcriptome. Using a multiples response method with a FDR (false discovery rate) < 0.05 we found that there were hundreds of genes differentially regulated across the different conditions (S1 Table).

We analyzed the hierarchical clustering and performed principal component analysis of mRNA expression profiles. Good reproducibility for each experimental point was demonstrated by the hierarchical clustering (Fig 1D). The only exception highlighted concerns a partial mismatch between OLF and CTRL, at 12h. In the principal component analysis, the PC1 axis clearly distinguished the experimental points referred to 6 and 12 hours while the PC2 clearly distinguished between control samples (CTRL, OLF and SO) and CSB suppressed cells (ASO) (Fig 1E). Interestingly the samples derived from CTRL-, OLF- and SO- treated cells clustered together while the ASO dramatically departed.

**Fig 2:**
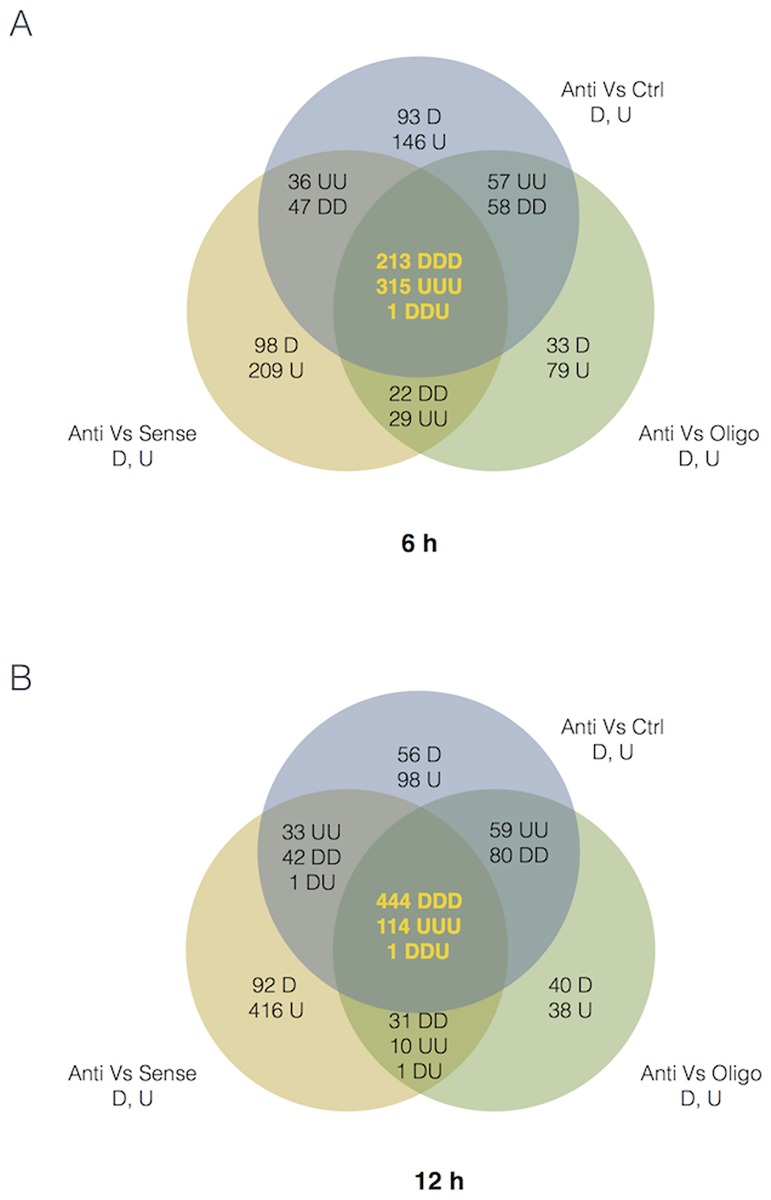
Venn diagrams showing pair-wise comparison between ASO and CTRL, ASO and OLF, finally ASO and SO at 6h and 12h. For intersections letters are in clockwise order of, for example DD for {Anti vs Ctrl} Intersect {Anti vs Oligo} means *first* D in {Anti vs Ctrl} and *second* D in {Anti vs Oligo}. Concerning the central intersection (yellow numbers) the clockwise order starts from Anti vs Ctrl. D means down-regulated and U up-regulated.

The gene list was initially filtered with the cut-off criteria FDR≤0.05 and absolute fold change |FC|≥1.5. Gene expression profiling revealed genes regulated uniquely by ASO, SO and OLF as compared to CTRL sample. To identify those genes whose expression level changed significantly and uniquely in response to CSB suppression we used a pair-wise comparison between ASO and CTRL, ASO and OLF, finally ASO and SO. In Fig 2 we represent the overlap between these sets of genes using Venn diagrams. Suppression of CSB affects the expressionof 528 (213 down-regulated and 315 up-regulated) genes at 6h and 558 (444 down-regulatedand 114 up-regulated) genes at 12h.

### Functional analysis of differentially expressed genes

To further understand the mechanisms by which CSB ablation induces apoptosis, differentially expressed genes (DEGs) obtained from the primary analysis were annotated by Gene Ontology (GO) to identify the Biological Process affected; enrichment analysis on up- and down-regulated genes was carried out using the DAVID web tool. A P-value < 0.05 and a Fold Enrichment (FE) value > 2 were chosen as cut off criteria. FE was calculated by dividing the frequency of specific gene cluster to the total frequency for each GO term.

Table 1 illustrates the FE of the major GO terms at 6h and 12h after the suppression of CSB. Altogether, gene ontology analysis revealed that responses to endoplasmic reticulum stress and to unfolded proteins, and induction of cell apoptosis in response to endoplasmic reticulum stress were ranked in the top cellular events being induced, and that the major components of endoplasmic reticulum stress-mediated apoptosis pathway were over-represented.

**Table 1:** 

**6h**			
**GO ID**	**Terms**	**Fold Enrichment**	**P-value**
**GO:1990440**	Positive regulation of transcription from RNA polymerase II promoter in response to endoplasmic reticulum stress	22.76	2.86E-02
**GO:0070059**	Intrinsic apoptotic signaling pathway in response to endoplasmic reticulum stress	15.86	7.67E-05
**GO:0030968**	Endoplasmic reticulum unfolded protein response	5.91	1.17E-02
**GO:0034620**	Cellular response to unfolded protein	5.75	1.54E-02
**GO:0035967**	Cellular response to topologically incorrect protein	5.33	3.30E-02
**GO:0044843**	Cell cycle G1/S phase transition	4.72	2.12E-02
**GO:0042594**	Response to starvation	4.53	3.42E-02
**GO:0072331**	Signal transduction by p53 class mediator	4.50	3.65E-02
**12h**			
**GO ID**	**Terms**	**Fold Enrichment**	**P-value**
**GO:0070059**	Intrinsic apoptotic signaling pathway in response to endoplasmic reticulum stress	12.58	2.95E-03
**GO:0006986**	Response to unfolded protein	5.42	2.73E-04
**GO:0030968**	Endoplasmic reticulum unfolded protein response	5.27	3.75E-02
**GO:0034620**	Cellular response to unfolded protein	5.13	4.89E-02
**GO:0035966**	Response to topologically incorrect protein	5.05	7.24E-04
**GO:0034976**	Response to endoplasmic reticulum stress	4.60	4.89E-05
**GO:0031396**	Regulation of protein ubiquitination	3.58	2.21E-02
**GO:0008380**	RNA splicing	3.16	9.38E-03

GO: Gene Ontology

**Fig 3:**
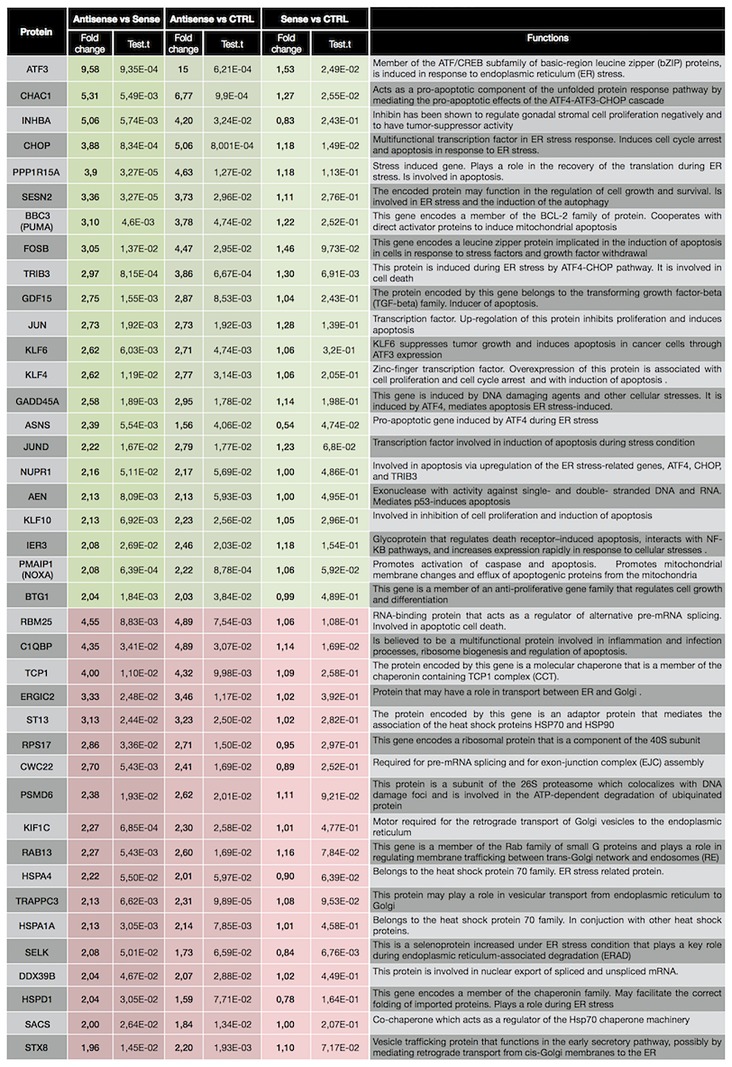
Filtered list of genes which showed a fold change > 2 (ASO vs SO) in the microarray analysis at 6h, and belonged to the top GO terms listed in Table 1.

**Fig 4:**
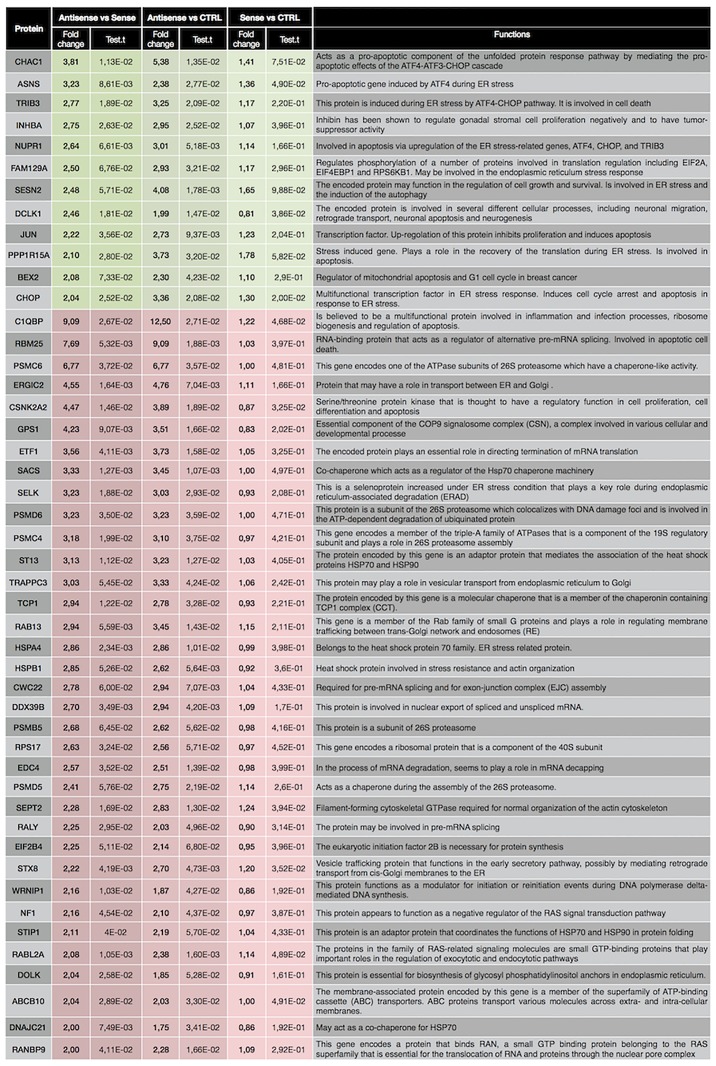
Filtered list of genes which showed a fold change > 2 (ASO vs SO) in the microarray analysis at 12h, and belonged to the top GO terms listed in Table 1.

With the aim to reduce the number of genes to focus our attention on, we further filtered the list to retain those genes which showed a fold change > 2 (ASO vs SO), and belonged to the top GO terms listed above. The list of genes so obtained is showed in Figs 3 and 4, for 6h and 12h, respectively. Activating transcription factor 3 (ATF3), a member of the ATF/cAMP response element binding protein (CREB) family of transcription factors induced by several stressors including ER stress, displays a dramatic increase (9.58 folds ASO vs SO) at 6h. Microarray data showed a 3.9 and 2 fold increase (ASO vs SO), at 6 and 12 h respectively, in CHOP, known to be the main mediator of ER stress-induced apoptosis. Along this line, CHAC1, PPP1R15A, BBC3 (PUMA), TRIB3 and NUPR1, pro-apoptotic components of unfolded protein response downstream of ATF3-CHOP cascade, showed a fold increase of 5.31, 3.9, 3.1, 2.97 and 2.16 respectively, at 6h. Moreover, with the exception of BBC3, the up-regulation of those genes is maintained also at 12h. In addition, general mediators of apoptosis and cell cycle arrest, such as INHBA, GADD45a and ASNS were also among the genes up-regulated in response to CSB suppression both at 6 and 12h.

Microarray data revealed a reduction in the gene expression of molecular chaperons and co-chaperons, including heat shock proteins (TCP1, HSPA4, HSPA1A, HSPD1, SACS, ST13, DNAJC21, STIP1, PSMC6, ST13) in either or both the times points. In addition, PSMD6, PSMD5, PSMB5, PSMC4 and PSMC6 subunits of the protease/chaperone complex proteasome 26S were also down regulated as were a number of splicing factors (RBM35, CWC22, C1QBP, RALY).

### Validation of microarray analysis data by quantitative real-time PCR

**Fig 5:**
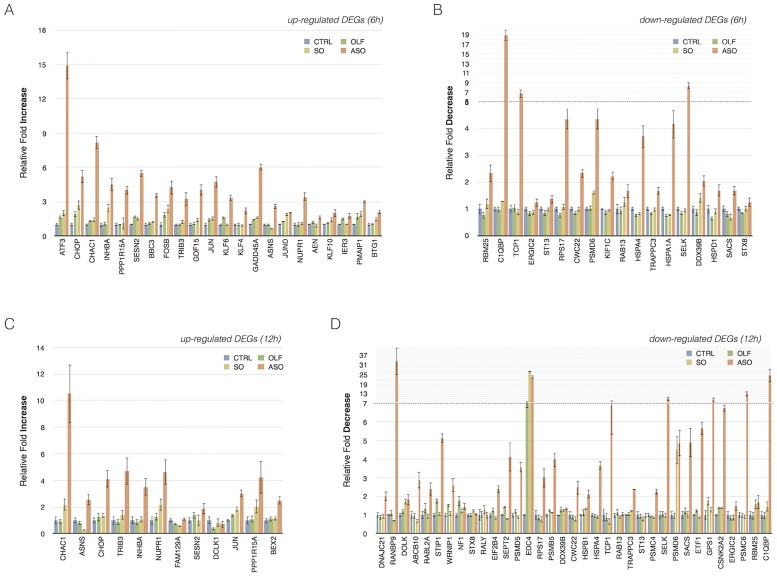
qPCR verification of DEGs. Graphs show relative fold change values of up-regulation (A and C) and down regulation (B and D) of selected genes. The results, normalized to β-actin, were averaged from values obtained by performing three technical replicates. The values are means ± SD.

**Fig 6:**
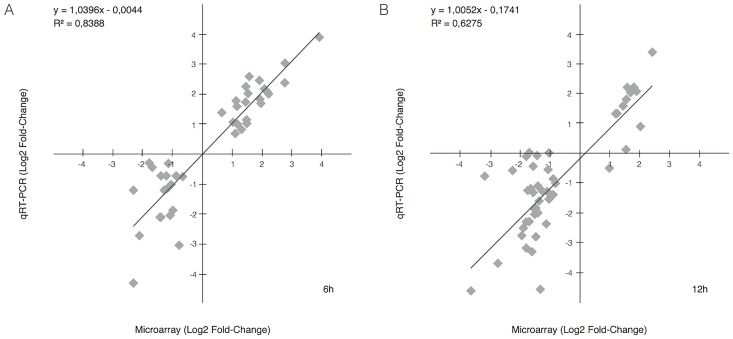
Validation of microarray data with qRT-PCR. Correlation between qRT-PCR and microarray analysis at 6h (A) and 12h (B). The correlation coefficients (R^2^) are 0.83 and 0.62, respectively.

As validation step of the microarray analysis results, relative expression of genes reported in Figs 3 and 4 was also measured by qRT-PCR using specific primer sets. Expression levels of each gene, either at 6 or 12 h, in OLF, SO and ASO treated cells have been compared to CTRL (Fig 5). As indicated by the graphs, for most of genes analysed, all the three controls samples (CTRL, OLF and SO) behave similarly while the CSB suppressed sample strongly diverges. To evaluate the correlation between microarray and qRT-PCR data, we calculated the log_2_ of fold changes of all selected genes in the CSB suppressed cells (ASO) relative to the control (CTRL) obtained from both the platform and tested the correlation between these two sets of data. Our results indicated a robust consistency between microarray and qRT-PCR data either at 6 and 12 h (*R*^*2*^ = 0.84, *P*< 0.0001 at 6h; *R*^*2*^ = 0.63, *P*< 0.0001 at 12h; Fig 6).

**Fig 7:**
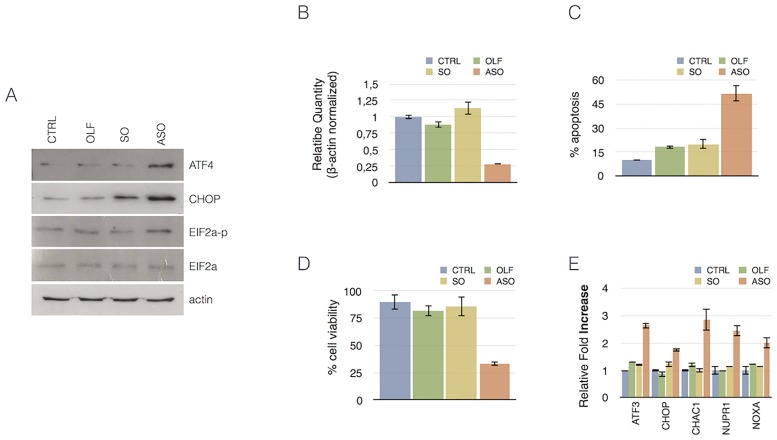
Western blotting demonstrating the significant increase of ATF4, CHOP and EIF2a-p after CSB knockdown. B) Graphs showing qRT-PCR analysis of CSB mRNA expression in SKNBE-2c cells, at 12h after the transfection. The results, normalized to β-actin, were averaged from values obtained by performing three technical replicates. The values are means ± SD. Graphs showing apoptosis (C) and cell viability (D) percentage in SKNBE-2c cells 48h after CSB ablation. The results were averaged from values obtained by performing three technical replicates. The values are means ± SD. E) Graphs show relative fold change values of up-regulation of selected genes in SKNBE-2c cells. The results, normalized to β-actin, were averaged from values obtained by performing three technical replicates. The values are means ± SD.

Next, to validate the above results, we analyzed by Western blotting the expression of proteins playing a key role in UPR. As showed in Fig 7A, the phosphorylation of eIF2α, which is triggered by the activation of ER resident transmembrane protein kinase PERK, one of the major sensor proteins which can detect the protein-folding imbalance generated by ER stress, is induced in CSB suppressed cells (ASO) compared to three different experimental controls. Accordingly, we observed that suppression of CSB also gave rise to an increased expression of ATF4, a master regulator of the UPR response and the pro-apoptotic transcription factor CHOP, both downstream targets of eIF2α phosphorylation.

Finally, with the aim to understand if suppression of CSB would cause ER stress also in other cell lines, we knocked down CSB in the neuroblastoma cells SKNBE-2c. ASO-induced suppression of CSB (Fig 7B) determined a strong induction of apoptosis (Fig 7C) and a correlated decrease of cell viability (Fig 7D). As illustrated in Fig 7E, RT-PCR revealed that suppression of CSB enhanced the expression of genes involved in the UPR response, as illustrated by the increased expression of ATF3, CHOP, CHAC1, NUPR1 and NOXA. Therefore the effect of CSB suppression on UPR response are not cell type specific.

## Discussion

Our findings reveal that HeLa cells are addicted to the elevated levels of CSB protein expression and that response to unfolded proteins and to endoplasmic reticulum stress is triggered upon CSB suppression. Furthermore, major components of UPR-mediated apoptosis pathway, including pro-apoptotic factors downstream of the ATF3-CHOP cascade, such as CHAC1, TRIB3, GADD34 and ASNS, are dramatically up-regulated and at the origin of the massive induction of apoptosis, displayed by HeLa cells upon CSB suppression. A similar behavior was also displayed when CSB was suppressed in neuroblastoma cells. One interesting question is why the outcome of the UPR response is only the activation of proapoptotic genes. Indeed UPR response also involves transcriptional induction of ER chaperone genes to enhance folding capacity and transcriptional induction of ER-associated degradation (ERAD) genes to increase protein degradation capacity [16–18]. All this responses are needed in order to maintain a productive ER protein folding environment and promote cell survival. It is only when these adaptive mechanisms fail to resolve protein folding defect that the UPR response tips the balance towards apoptosis by up regulating the ATF3-CHOP cascade [19–21].

In contrast, we observed that all these adaptive (pro-survival) mechanisms are down-regulated, upon CSB suppression. Chaperons folding protein-coding genes, such as TCP1, HSPA1A, HSPA4 and HSPD1, are down-regulated as well. Along the same line, expression of SELK, involved in ERAD, is also down-regulated. Down-regulation of mediators of UPR pro-survival pathway and up-regulation of mediators of UPR pro-death pathway upon CSB suppression might be explained in the context of transformed cells (HeLa and SKNBE-2c). Activation of pro-survival UPR response is an adaptive survival strategy that cancer cells adopt to deal with the increasing levels of ER stress determined by their increased anabolism (increased protein production, mTOR activation) and hostile environmental (hypoxia, nutrient deprivation, reactive oxygen species (ROS) and redox changes) [22]. Activation of pro-apoptotic genes upon CSB suppression conjointly with down-regulation of adaptive pro-survival genes let us speculate that ablation of CSB determines, in cancer cells, an increase of the (pre-existing) ER stress that tip the balance from pro-survival towards apoptosis signalling.

This opens a new scenario for the role played by CSB in tumour biology. What is CSB role in limiting ER stress? Is CSB involved in protein folding? These are new and exciting areas that warrant investigation.

Hallmarks of cancer are prone to induce stress at various levels. Oxidative stress, hypoxia and ER stress are something that the cancer are to deal with [23–27]. Furthermore, all these processes are interdependent. Therefore, cancer cells have to cope with multiple stress conditions at once, all prone to induce apoptosis.

It is known that CSB protein counteracts oxidative stress detrimental effect. Some authors have recently shown that CSB-mutated cells have increased levels of intra-mitochondrial ROS suggesting that CSB might behave as an electron scavenger in the mitochondria and that its absence leads to increased oxidative damage [28]. We showed that CSB also favours pro-survival HIF-1 induced response to attenuate the detrimental effect of hypoxia condition [12], including increased generation of mitochondrial ROS [29].

Both oxidative stress and hypoxia, if not adequately contrasted, are known to induce ER stress. ROS can oxidize cysteine residues, leading to excessive disulphide bond formation in proteins and consequent folding alteration [30]. Hypoxia leads to a deficit in the production of ATP and consequently a reduced chaperon activity in the ER that results in impaired protein folding and ER stress [31]. Lastly, it is emerging that CSB together with CSA is associated to the ubiquitin/proteasome system [32–33]. It might be that a reduced presence of CSB could somehow affect the efficiency of protein degradation.

We can speculate that over expression of CSB, as observed in tumours [15], is an adaptive mechanism that the cancer cell develops to counteract hypoxia condition, ROS overproduction and to limit the presence of incorrectly folded proteins, to reduce ER stress.

It has been described that oncogene and tumour suppressor gene mutations might inhibit ER-stress induced apoptosis machinery [27]. Our work, instead, confirms that UPR activation induced cell death is intact in at least some tumour cells and that ER stress and UPR activation may offer a target for combination therapy.

Finally, as well known, CSB mutations result in Cockayne syndrome, a premature aging syndrome characterized by a severe neurodegenerative clinical picture. The importance of the UPR in the process is observed in a number of neurodegenerative diseases including amyotrophic lateral sclerosis, Parkinson’s disease, Huntington’s disease, Alzheimer’s disease and demyelinating neurodegenerative autoimmune diseases such as multiple sclerosis [34–35], which introduces challenges to study the functional significance of ER stress in the pathogenesis of Cockayne syndrome.

## Material and methods

### Cell culture and gene expression silencing

Cell lines were grown in DMEM (Hela) or a mixture of Eagle’s-MEM/F12 (SKNBE-2c) containing 10% FCS and Gentamicin in a 5% CO_2_ humidified atmosphere at 37°C.

For transfection procedure Thermo Fisher Scientific Protocol for Oligofectamine reagent was used. Briefly the day before transfection 1x10^5^ cells were plated in 6-well dishes using medium without antibiotics. Immediately before transfection the medium was replaced with Optimem and oligonucleotides (200 nM final concentration) were delivered using Oligofectamine transfection reagent. The cells were transfected with ASO or SO or simply exposed to the transfectant reagent (OLF). Four hours after transfection Optimem was replaced with fresh complete medium. Total RNA was extracted at 2h or 8h after medium adding. Sense oligonucleotides were the reverse of the antisense sequence. Oligonucleotide sequences are available on request.

### Cell viability assay

Cell viability was evaluated using MTT [3-(4,5-dimethylthiazol-2-yl)-2,5-diphenyl-2H-tetrazolium bromide] cell proliferation assay. Cells were plated in 96-well plates one day before oligonucleotides transfection. MTT was added to each well (0.5 mg/ml) 48h after transfection. After incubation for 3 h at 37°C, the supernatant was replaced with 150ul of solution (10% SDS, 0.6% acetic acid in DMSO) to dissolve the formazan crystals and produce a purple solution. Optical density measurements were obtained using a scanning spectrophotometer DTX 880 Multimode Detector (Beckman Coulter). The readings were made using a 630 nm (background) and a 570 nm filter. The assays were conducted in triplicate for each condition.

### Apoptosis analysis

Cells were plated in 6-well plates one day before oligonucleotides transfection as described above; 48 h later the oligonucleotides transfection, a combination of Fluorescein Diacetate (FDA; 15 g/ml), Propidium Iodide (PI; 5 g/ml) and Hoechst (HO; 2 g/ml) were used to differentiate apoptotic and necrotic cells from viable cells. FDA and HO are vital dyes that stain the cytoplasm and nucleus of the viable cells, respectively. The necrotic and the late stage of apoptotic cells are readily identified by PI staining. Cells in the early phase (viable––HO stained) and late phase (dead––PI stained) of apoptosis displayed the characteristic pattern of chromatin fragmentation. Approximately 2000 randomly chosen cells were microscopically analyzed to determine apoptosis levels.

### RNA isolation, amplification, labelling and hybridization of microarrays

Total RNA was isolated from HeLa cells using Absolutely RNA Miniprep kit (Agilent Technologies). Quality and Integrity of each sample was checked using Agilent BioAnalyzer 2100 (Agilent RNA 6000 nano kit): samples with a RNA Integrity Number (RIN) index lower than 8.0 were discarded. Aliquots from the same RNA sample, prepared (and pooled) from 3 different experiments were used for the hybridizations to reduce the experimental variability.

All the experimental steps for gene expression profiling, involving the labelling, hybridization and washings of the samples, were done following the one color microarray Agilent protocol (Agilent Technologies, Inc, Santa Clara, CA, USA). CTRL, OLF, SO and ASO samples were labelled with Cy3 and hybridized on Agilent SurePrint G3 Human GE 8x60K Microarrays (grid ID 034494).

### Scanning, feature extraction and analysis

Post-hybridization image acquisition was accomplished using the Agilent scanner G2564B, equipped with lasers at 532 nm. Data extraction from the Agilent scanner images was accomplished by Feature Extraction software. Data filtering and analysis were performed using R-Bioconductor and Microsoft Excel. All the features with the flag gIsWellAboveBG = 0 in raw data files (too close to background) were filtered out and excluded from the following analysis. Filtered data were normalized to the 75th percentile.

Data were further filtered by excluding mRNA probes with a coefficient of variation |Dev.st/Mean|>5% in Log2 expression values between biological replicates, across all samples, to obtain a final selection 17703 transcripts.

Differentially expressed genes were selected by a combination of fold change and moderated T-test thresholds by the R-Bioconductor tool Limma (p-value<0.05; |Log2 fold-change ratio| >0.56 equivalent to 1.5 fold in linear scale).

Hierarchical Clustering (HCL) and Principal Component Analysis (PCA) were used to show overall differences between ASO and control samples in transcriptional profiles (Fig 1D and 1E). The analysis of over- and under- represented functional in gene lists annotations was performed using the chart and cluster algorithms of DAVID web tool [36]. The whole microarray dataset is public and available from the Gene Expression Omnibus database (https://www.ncbi.nlm.nih.gov/geo).

### Retrotranscription and real-time quantitative PCR

Real-time quantitative PCR was used to verify CSB silencing and the accuracy of microarray data. Total RNA was reverse transcribed for single-stranded cDNA using oligo-(dT)_18_ primer of First Strand cDNA Synthesis kit (Fermentas). Real-time quantitative PCR was carried out with SYBR green master mixture (Promega) using Mx3005P Real-Time PCR system (Agilent). For quantification of gene expression changes, the Ct method was used to calculate relative fold changes normalized against the housekeeping gene beta actin. To obtain more reliable results, all reactions were performed in triplicate. Primers sequences are available on request.

Concerning microarray data validation, the choice of the mRNA species analyzed by qRT-PCR was made according to the criteria listed in Result.

## Supporting information

S1 Table Differentially expressed mRNA transcripts. The 6 lists correspond to the same 6 comparisons shown in the Venn diagrams of Fig 2. Transcripts were selected, on filtered dataset (see Methods) by a combination of fold change and moderated T-test thresholds by the R-Bioconductor tool Limma (p-value<0.05; |Log2 fold-change ratio| >0.56 equivalent to 1.5 fold in linear scale).
(XLSX)
